# BEHAVIOR-RELATED RISK FACTORS AND WORKING LIFE EXPECTANCY IN ENGLAND

**DOI:** 10.1093/geroni/igad104.1048

**Published:** 2023-12-21

**Authors:** Paola Zaninotto

**Affiliations:** University College London, London, England, United Kingdom

## Abstract

Over the past decades, the UK has introduced pension and labour market reforms that aimed at increasing the length of working life among the older working population. Behaviour-related risk factors such as low physical activity level, alcohol consumption, smoking as well as obesity are associated with labour market participation and early retirement. However, the extent to which the co-occurrence of these behaviour-related risk factors predict how long people stay in employment (working life expectancy) has not yet been investigated. We used a large nationally representative sample of older people in England participants of the English Longitudinal Study of Ageing (ELSA), from 2002 to 2018. We applied multistate life table models to repeated measures to estimate sex-specific working life expectancy from the age of 50. We considered the following behaviours: smoking, daily alcohol consumption, being physically inactive and being obese. At the age of 50, men with no behaviour-related risk factors could expect to work on average 11 years (95%CI:10.96; 11.44) longer (9 for women, 95%CI:8.98;9.44), whereas men with 2+ behaviour-related risk factors could expect to work on average 9.2 additional years (95%CI:9.19; 9.85) (7.4 for women, 95%CI:7.3; 7.8). Men and women engaging in one behaviour-related risk factor had slightly higher lower working life expectancy than those not engaging in any behaviour-related risk factors.

